# Changing Indications and Socio-Demographic Determinants of (Adeno)Tonsillectomy among Children in England – Are They Linked? A Retrospective Analysis of Hospital Data

**DOI:** 10.1371/journal.pone.0103600

**Published:** 2014-08-11

**Authors:** Elizabeth Koshy, Alex Bottle, Joanna Murray, Mike Sharland, Sonia Saxena

**Affiliations:** 1 Department of Primary Care and Public Health, Imperial College London, London, United Kingdom; 2 Doctor Foster Unit, Imperial College London, London, United Kingdom; 3 Paediatric Infectious Diseases Unit, St George's Hospital NHS Trust, London, United Kingdom; Peking Union Medical College, China

## Abstract

**Objective:**

To assess whether increased awareness and diagnosis of obstructive sleep apnoea syndrome (OSAS) and national guidance on tonsillectomy for recurrent tonsillitis have influenced the socio-demographic profile of children who underwent tonsillectomy over the last decade.

**Method:**

Retrospective time-trends study of Hospital Episodes Statistics data. We examined the age, sex and deprivation level, alongside OSAS diagnoses, among children aged <16 years who underwent (adeno)tonsillectomy in England between 2001/2 and 2011/12.

**Results:**

Among children aged <16 years, there were 29,697 and 27,732 (adeno)tonsillectomies performed in 2001/2 and 2011/12, respectively. The median age at (adeno)tonsillectomy decreased from 7 (IQR: 5–11) to 5 (IQR: 4–9) years over the decade. (Adeno)tonsillectomy rates among children aged 4–15 years decreased by 14% from 350 (95%CI: 346–354) in 2001/2 to 300 (95%CI: 296–303) per 100,000 children in 2011/12. However, (adeno)tonsillectomy rates among children aged <4 years increased by 58% from 135 (95%CI: 131–140) to 213 (95%CI 208–219) per 100,000 children in 2001/2 and 2011/2, respectively. OSAS diagnoses among children aged <4 years who underwent surgery increased from 18% to 39% between these study years and the proportion of children aged <4 years with OSAS from the most deprived areas increased from 5% to 12%, respectively.

**Conclusions:**

(Adeno)tonsillectomy rates declined among children aged 4–15 years, which reflects national guidelines recommending the restriction of the operation to children with more severe recurrent throat infections. However, (adeno)tonsillectomy rates among pre-school children substantially increased over the past decade and one in five children undergoing the operation was aged <4 years in 2011/12.The increase in surgery rates in younger children is likely to have been driven by increased awareness and detection of OSAS, particularly among children from the most deprived areas.

## Introduction

(Adeno)tonsillectomy rates in children have declined in the United Kingdom (UK), many other Western European countries and the United States (US) over recent decades [1] [2], in the wake of strong evidence and national guidance recommending that (adeno)tonsillectomy should be reserved for children with severe recurrent acute throat infections (ATI) [3], [4]. Despite declining rates, (adeno)tonsillectomy remains one of the most commonly performed operations in children worldwide [5] [6] [7]. Although the most frequent indication for (adeno)tonsillectomy has been recurrent tonsillitis, a growing number of operations are reportedly performed for the sleep-disordered breathing (SDB) spectrum, in particular, obstructive sleep apnoea syndrome (OSAS), which most commonly affects pre-school children (aged <5 years) [6], [7]. In the US, there has been a shift towards performing (adeno)tonsillectomy in younger children and an increasing number of pre-school children have undergone surgery for the SDB spectrum over recent years with low complication rates [7], [8]. A small number of studies and reviews suggest similar trends have occurred in the UK and other European countries and are consistent with evidence of increased awareness, diagnosis and surgical intervention for the SDB spectrum [9] [10]. A small Scottish study found an association between deprivation and (adeno)tonsillectomy, particularly operations performed for symptoms related to SDB or OSAS [9], whilst a Canadian observational study of children with and without OSAS, found that those with OSAS were more likely to reside in disadvantaged areas [11].

There have been contrasting findings relating to a potential association between socio-economic status (SES) and (adeno)tonsillectomy operations [12] [13] [14]. A recent pragmatic trial based in the North of England and Scotland and a US study did not find an association with SES [12] [13]. However, marked deprivation and geographical gradients have been reported in other studies in England, Scotland and Canada [14] [15], [16] [17]. Deprivation is known to be a determinant of poor health in children although the precise causal mechanisms are not fully understood [18]. A potential contributory factor for higher tonsillectomy rates among more deprived populations may be higher prevalence rates of smoking in households, which is associated with increased upper respiratory infections, including tonsillitis [19]. Lower SES has been found to be an independent risk factor for the SDB spectrum in children and also for respiratory disease and group A streptococcal infections, all of which can increase the need for (adeno)tonsillectomy [12], [20] [21] [22]. Despite evidence that childhood asthma hospital admissions have declined in England following smoke-free legislation [23], respiratory infections including tonsillitis admission rates have increased [24]. Little is known about how these socio-economic differentials in tonsillitis incidence and the SDB spectrum have affected the socio-demographic profile of children who actually undergo (adeno)tonsillectomy.

Our aim was to investigate changes in the socio-demographic profile of children who underwent (adeno)tonsillectomy over the last decade. We hypothesised that increased awareness and diagnosis of OSAS during the last decade has influenced the socio-demographic profile of children who undergo (adeno)tonsillectomy. Specifically, that this change may have led to an increase in (adeno)tonsillectomy rates, particularly among younger children from the most deprived areas. We examined the age, sex and deprivation level, alongside OSAS diagnoses, among children undergoing (adeno)tonsillectomy in England, using national data between 2001/2 and 2011/12.

## Materials and Methods

The Hospital Episodes Statistics (HES) database captures all NHS patient activity in England (www.hesonline.org.uk). The data contain demographic, administrative and clinical information including procedures and operations. Operations performed in hospital are coded using the OPCS-4 system (Office of Population, Censuses and Surveys: Classification of interventions and procedures, 4th Revision). We studied all children aged <16 years who had tonsillectomy as their primary operation recorded in UK financial years 2001/2 (April 2001 to March 2002) and 2011/12 (April 2011 to March 2012). The tonsillectomy codes *(F34)* were based on those included in a UK Department of Health report on ‘Trends in Children's Surgery’ [5]. We included all tonsillectomy operations which were performed alone and also as part of adeno-tonsillectomy.

**Figure 1:**
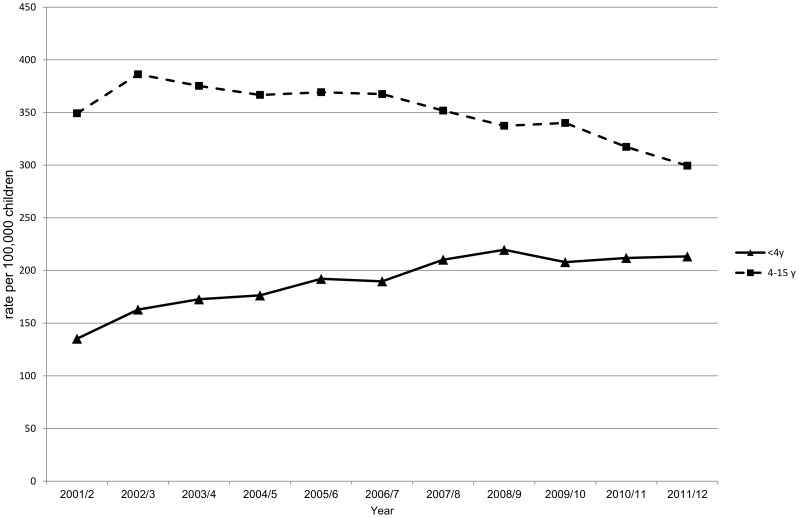
Trends in (adeno)tonsillectomy rates by age-group (<4 years and 4–15 years), 2001/2 to 2011/12.

We calculated (adeno)tonsillectomy rates per 100,000 children by dividing the total number of operations by the Office for National Statistics (http://www.statistics.gov.uk/default.asp) mid-year child population estimates. We investigated how rates varied according to age, using four age groups: <4, 4–7, 8–11 and 12–15 years. These age categories have been used in previous (adeno)tonsillectomy studies and so aid comparison [13]. We also calculated crude annual age group specific (adeno)tonsillectomy rates for children aged <4 years and 4–15 years (Figure 1). HES does not include the reason for surgery. Therefore, these age categories are a proxy measure for the likely indication. OSAS most commonly affects pre-school children and this is the group in which the highest (adeno)tonsillectomy rates are performed for this indication [7], [9]. Older children are more commonly operated on for recurrent throat infections, particularly as *group A beta haemolytic streptococcus* is most prevalent among children aged 5–15 years [25]. Previous (adeno)tonsillectomy studies for recurrent throat infections have also focussed on children aged 4–15 years [13]. We examined the deprivation level of children who underwent (adeno)tonsillectomy by age group, using the Carstairs index for deprivation level, which uses the patient's residential postcode and is divided into population-weighted quintiles (with 1 representing the least deprived and 5, the most deprived) [26]. We also examined the distribution of (adeno)tonsillectomies by sex.

Finally, we identified children who had a ‘sleep apnoea’ ICD-10 code (*G37.3*) within one of the HES diagnostic fields during the index admission for (adeno)tonsillectomy. This acted as a proxy measure for awareness and detection of OSAS. We calculated the proportion of children with an OSAS diagnosis among those aged <4 years who underwent (adeno)tonsillectomy in 2001/2 and 2011/12. We also compared the proportion of children with the combination of OSAS and who lived in the most deprived areas (quintile 5) in both these study years. We repeated this analysis for children aged 12–15 years, as a reference category.

All rates were calculated with associated 95% confidence intervals (CI). We used the χ^2^ test to investigate the difference between categorical groups and the χ^2^ test for linear trend to assess potential linear relationships between two variables. We tested for a linear relationship between age group and sex for (adeno)tonsillectomy in 2001/2 and 2011/12, based on observations in a recent study of children referred for (adeno)tonsillectomy [13]. We considered two-sided probability values less than 0.05 to be statistically significant. Statistical analysis was performed using Stata/SE version 11.1 (www.stata.com).

### Ethics statement

This study was approved under Section 251 (formerly Section 60) granted by the National Information Governance Board for Health and Social Care (NIGB, formerly the Patient Information Advisory Group). We also have approval for using these data for research from the South East Research Ethics Committee. The patient records were anonymised and de-identified prior to analysis.

## Results

There were 29697 and 27732 (adeno)tonsillectomies performed in English NHS hospitals among children aged <16 years in 2001/2 and 2011/12, respectively.

The median age at (adeno)tonsillectomy declined from 7 years (IQR: 5–11) to 5 years (IQR: 4–9) over the decade. Children aged <4 years represented 11% (3132/29697) of all (adeno)tonsillectomy operations in 2001/2 and this doubled to 21% (5720/27732) in 2011/12. The percentage of children undergoing (adeno)tonsillectomy who were girls fell slightly from 53% (15765/29697) in 2001/2 to 50% (13809/27732) in 2011/12 (Tables 1 and 2). There was a linear relationship between age group and sex for (adeno)tonsillectomy in 2001/2 and 2011/12 for girls and a corresponding inverse linear relationship between these two variables for boys in each of these study years (P<0.001 for linear trend) in both 2001/2 and 2011/12 (Tables 1 and 2). Girls were increasingly likely to undergo (adeno)tonsillectomy with increasing age whilst boys were less likely to undergo surgery with increasing age in both study years.

**Table 1:** (Adeno)tonsillectomy by age group and sex in 2001/2 (N = 29697).

	Age (years)				Total	P value*
SEX	<4	4 to 7	8 to 11	12 to 15		
**Male (n)**	2015	7578	2506	1833	13932	
**%**	64	54	39	30	47	
**Female(n)**	1117	6435	3953	4260	15765	P<0.001
**%**	36	46	61	70	53	
**Total (n)**	3132	14013	6459	6093	29697	
**%**	100	100	100	100	100	

Data source: Hospital Episodes Statistics data.

*χ^2^ test for linear trend.

**Table 2:** (Adeno)tonsillectomy by age group and sex in 2011/12 (N = 27732).

	Age (years)				Total	P value*
SEX	<4	4 to 7	to 11	12 to 15		
**Male (n)**	3600	7322	1837	1164	13923	
**%**	63	55	40	28	50	
**Female(n)**	2120	6029	2722	2938	13809	P<0.001
**%**	37	45	60	72	50	
**Total (n)**	5720	13351	4559	4102	27732	
**%**	100	100	100	100	100	

Data source: Hospital Episodes Statistics data.

*χ^2^ test for linear trend.

The overall (adeno)tonsillectomy rate among children aged <16 years was 300 (95% CI: 296–303) per 100,000 children in 2001/2 and declined to 277 (95% CI: 273–280) per 100,000 in 2011/12. (Adeno)tonsillectomy rates among children aged <4 years increased by 58% from 135 (95% CI: 131–140) to 213 (95% CI: 208–219) per 100,000 children in 2001/2 and 2011/2, respectively (Figure 1). By contrast, (adeno)tonsillectomy rates among children aged 4–15 years decreased by 14% from 350 (95% CI: 346–354) to 300 (95% CI: 296–303) per 100,000 children (Figure 1). Among children within the 4–15 year age category, the largest absolute and relative reduction in (adeno)tonsillectomy rates over the decade were seen among children aged 12–15 years (Table 3). There was an approximately linear decline in (adeno)tonsillectomy rates among children aged 4–15 years between 2002/3 and 2011/12 and, conversely, an almost linear increase among children aged <4 years over this time frame (Figure 1). Notably, for all the age-group specific (adeno)tonsillectomy rates, the associated 95% confidence intervals are narrow and do not overlap, which shows statistical significance (Table 3).

**Table 3:** (Adeno)tonsillectomy rates by age group, 2001/2 and 2011/12.

Age group (years)	(Adeno)tonsillectomy rate 2001/2 (95% CI)	(Adeno)tonsillectomy rate 2011/12 (95% CI)	Rate ratio (95% CI)
**<4**	135 (131 to 140)	213 (208 to 219)	1.58 (1.51–1.65)
**4 to 7**	571 (561 to 580)	536 (527 to 545)	0.94 (0.92–0.96)
**8 to 11**	249 (243 to 255)	196(190 to 201)	0.79 (0.76–0.82)
**12 to 15**	238 (232 to 245)	162 (157 to 167)	0.68 (0.65–0.71)

Rates per 100,000 children.

Data source: Hospital Episodes Statistics data.

Children aged <16 years who underwent (adeno)tonsillectomy were 2.2 times as likely to be from the most deprived areas (quintile 5) than from the least deprived areas (quintile 1) in 2001/2 and 1.8 times as likely in 2011/12 (Tables 4 and 5). Children aged <4 years who underwent (adeno)tonsillectomy were 1.5 times as likely to be from the most deprived areas compared with the least deprived areas in 2001/2 and 1.6 times as likely in 2011/12. Furthermore, among children aged <4 years who underwent (adeno)tonsillectomy, those from the most deprived areas increased from 35 (95% CI: 32–37) to 55 (95% CI: 53–58) per 100,000 children between 2001/2 and 2011/12, respectively. This absolute increase in (adeno)tonsillectomy rate was larger than that for any other deprivation level or age group (Tables 4 and 5). Conversely, among children in the 4–15 year age range, the ratio of those who underwent surgery from the most deprived areas compared with the least deprived areas decreased between these study years and this was most pronounced among children aged 12–15 years (Tables 4 and 5). Our deprivation level data were >99% complete.

**Table 4:** Distribution of children who underwent (adeno)tonsillectomy by deprivation level in 2001/2 (N = 29653)*.

Age group		Carstairs deprivation level (quintiles)				
		1 (least deprived)	2	3	4	5 (most deprived)
	n	Rates per 100,000 children (95% CI)				
**<4 years**	3127	24	22	25	30	35
		(22–26)	(20–24)	(23–27)	(28–32)	(32–37)
**4–7 years**	13991	72	84	100	138	177
		(69–76)	(81–88)	(96–104)	(133–142)	(172–182)
**8–11 years**	6449	33	36	46	59	74
		(31–36)	(34–38)	(44–49)	(56–62)	(71–78)
**12–15 years**	6086	31	39	45	56	66
		(29–34)	(37–42)	(43–48)	(53–59)	(63–70)
**All ages**	29653	40	46	54	71	88
**<16 years**		(39–42)	(44–47)	(53–56)	(69–73)	(87–90)

Data source: Hospital Episodes Statistics data.

*Deprivation data >99% complete.

**Table 5:** Distribution of children who underwent (adeno)tonsillectomy by deprivation level in 2011/12 (N = 27610)*.

Age group		Carstairs deprivation level (quintiles)				
		1 (least deprived)	2	3	4	5 (most deprived)
	n	Rates per 100,000 children (95% CI)				
**<4 years**	5674	35	35	42	45	55
		(32–37)	(33–38)	(39–44)	(42–47)	(53–58)
**4–7 years**	13296	77	82	96	119	159
		(74–81)	(79–86)	(92–100)	(115–124)	(154–164)
**8–11 years**	4545	30	32	36	42	55
		(28–33)	(30–34)	(33–38)	(39–45)	(52–58)
**12–15 years**	4095	26	27	30	37	41
		(24–28)	(25–30)	(28–33)	(35–40)	(38–43)
**All ages**	27610	42	44	51	61	77
**<16 years**		(41–43)	(43–46)	(50–52)	(59–62)	(76–79)

Data source: Hospital Episodes Statistics data.

*Deprivation data >99% complete.

We found that OSAS diagnoses doubled (from 18.4 to 39.0%, P<0.001) between 2001/2 and 2011/12 among children aged <4 years who underwent (adeno)tonsillectomy (Table 6). The proportion of children with OSAS from the most deprived areas also doubled (from 5.1 to 11.8%, P<0.001). OSAS diagnoses among children aged 12–15 years who underwent surgery increased from 0.7 to 4.5% (P<0.001) but these percentages were markedly lower, in both years, compared with the younger age group (Table S1). The proportion from the most deprived areas decreased in the older age group. Finally, children aged 12–15 years with OSAS from the most deprived areas increased but the percentages were very low in both years.

**Table 6:** Obstructive sleep apnoea syndrome and deprivation among children who underwent (adeno)tonsillectomy (aged <4 years).

Children aged <4 years who underwent (adeno)tonsillectomy									
	OSAS diagnoses			Living in most deprived areas			OSAS AND living in most deprived areas		
Year	n	%	P value*	n	%	P value*	n	%	P value*
**2001/2 (n = 3127)**	575	18.4	**P<0.001**	805	25.7	P = 0.635	161	5.1	**P<0.001**
**2011/12 (n = 5674)**	2213	39.0		1487	26.2		670	11.8	

Data source: Hospital Episodes Statistics data.

*χ^2^ test.

OSAS – obstructive sleep apnoea syndrome.

Deprivation data >99% complete.

## Discussion

### Main findings

(Adeno)tonsillectomy rates increased by 58% among children aged <4 years and OSAS diagnoses in this subgroup doubled over the decade. In 2011/12, one in five (adeno)tonsillectomy operations in children were performed on those aged <4 years and this doubled from a decade earlier. By contrast, (adeno)tonsillectomy rates among children aged 4–15 years declined by 14%. Twice as many children aged <16 years who underwent (adeno)tonsillectomy were from the most deprived areas compared with those from the least deprived areas in both study years.

### (Adeno)tonsillectomy rates among young children

(Adeno)tonsillectomy rates increased by 58% among children aged <4 years between 2001/2 and 2011/2 driven by increasing OSAS diagnoses that rose from 18% to 39% between these study years. Our findings flank the proportion observed in a UK national audit of 33% among children aged <5 years in 2003/4 [27]. The rise is also consistent with a Scottish study, which reported ambulatory (adeno)tonsillectomies in children for the SDB spectrum increased from 26% to 55%, between 2001 and 2011, respectively, and SDB was the most common indication for (adeno)tonsillectomy among pre-school children (aged <5 years) [9]. A likely explanation is that this is a consequence of increased awareness and diagnostic rates of SDB among family practitioners and otolaryngologists [28] [29]. A recent study in England showed a four-fold increase in childhood obesity-related admission rates during the last decade, from 93 to 414 per million children and many of these admissions were for sleep apnoea [30]. Therefore, rising procedures for SDB may be partly driven by increasing levels of childhood obesity, particularly among those with lower SES [31], as there is an association between obesity and deprivation, and obesity is a recognised independent risk factor for OSAS [32]. We observed that the largest absolute increase in (adeno)tonsillectomy rates among children aged <4 years over the decade was in children from the most deprived areas. Thus there could be a three-way relationship between OSAS, obesity and deprivation for children who undergo (adeno)tonsillectomy. Our findings showed a doubling of the prevalence of OSAS diagnoses among children aged <4 years who underwent (adeno)tonsillectomy over the decade. The proportions of children from the most deprived areas and those with OSAS who were from the most deprived areas also increased. Therefore, this provides some evidence supporting our original hypothesis of a two-way relationship between OSAS and deprivation with (adeno)tonsillectomy, whereby OSAS diagnoses have increased in young children over the decade and that rise has predominantly affected those from the most deprived areas. However, there were virtually no children with a record of obesity (ICD-10 code *E66*) within our data to investigate a three-way relationship.

There has been a shift from infection to SDB for the indication for surgery among younger children in the US, whereas infection was more common a reason in older children [7]. That US study identified that obstructive symptoms were the primary indication for (adeno)tonsillectomy in 91.8% of patients aged 0–3 years [7], which is markedly higher than that reported in the UK [9] [27]. These differences may reflect that there are national guidelines for the diagnosis and management of OSAS in children in the US [32] but not in the UK. These US guidelines also highlight the need for a greater evidence base on screening for and diagnosing OSAS, evaluating different treatment methods in otherwise healthy children and investigating the role of obesity.

### (Adeno)tonsillectomy rates among older children

The decline in (adeno)tonsillectomy rates among children aged 4–15 years may be a consequence of UK national guidelines and also a Cochrane systematic review, which were both first published in 1999, and highlighted that (adeno)tonsillectomy should be reserved for those who are more severely affected by recurrent throat infections [3], [4], particularly as older children are more commonly referred for throat infections than younger children [7], [12]. Our rates per 100,000 children are considerably lower than those reported in the US. A US study of ambulatory (adeno)tonsillectomy operations in 2006, found the (adeno)tonsillectomy rate was 91.3 (95% CI: 46.6–136.0) per 10,000 children among children aged 7–12 years and 102.9 (95% CI: 66.7–139.1) per 10,000 children among children aged <7 years [12]. However, the US has consistently had higher rates of (adeno)tonsillectomy among children over recent decades [2]. In 2009, UK otolaryngologists issued a position paper raising concerns that the UK currently has one of the lowest (adeno)tonsillectomy rates in Europe, which they suggested has contributed to a major increase in serious tonsillitis infections and associated complications, such as peritonsillar abscess (PTA) [1]. Although our recent study found no evidence of an increase in severity of acute throat infection (ATI) admissions or PTA rates to justify decreasing the threshold for (adeno)tonsillectomy for recurrent sore throats, it is important to continue to monitor (adeno)tonsillectomy rates, following the wider dissemination of this position statement, to see if the rates in older age groups start to increase again [24].

The ratio of children aged 4–15 years from the most deprived areas compared with the least deprived areas declined between 2001/2 and 2011/12, which suggests narrowing of inequalities for (adeno)tonsillectomy potentially indicated for recurrent throat infections.

### Deprivation and (adeno)tonsillectomy

Our findings of a deprivation gradient in (adeno)tonsillectomy rates are consistent with those reported in the Chief Medical Officer's 2005 annual report (which studied HES data from 2004/5) and highlighted a reversal in (adeno)tonsillectomy trends between social classes over the last century. The report postulated that more affluent parents may be better informed and empowered to influence their child's health and treatment decisions compared with more disadvantaged parents [14]. However, our findings for deprivation are in contrast to those reported by Lock *et al* who found no association between SES based on parental occupation and (adeno)tonsillectomy [13]. Only 1015 children were eligible to enter the study to potentially undergo (adeno)tonsillectomy, which is substantially lower than a population-level sample using HES. The aforementioned US study also found similar ambulatory (adeno)tonsillectomy rates among children who were covered by Medicaid compared with private insurance [12]. Two older studies, a Scottish study from 1990 and a Canadian study from 1996–2000, found that whilst (adeno)tonsillectomy rates were significantly higher in areas with greater deprivation levels, grommet insertion rates were significantly lower [16] [17]. This suggests that there is not necessarily a consistent deprivation gradient for all paediatric conditions and operations and furthermore, that a deprivation gradient was observed 20 years ago [16], [17] [33]. Hence we recommend that the reasons for this persisting deprivation gradient for (adeno)tonsillectomy rates warrant further investigation.

(Adeno)tonsillectomies performed in the private sector should ideally be included in future analysis to determine if a deprivation gradient persists. If their inclusion still supports these findings, qualitative research, in the form of questionnaires and semi-structured interviews, may help to examine why a deprivation gradient exists. It would also be of interest to see if a deprivation gradient exists for other operations.

### Sex differences for (adeno)tonsillectomy

Our observation that older girls were more likely to have an (adeno)tonsillectomy than boys and that younger boys were more likely to undergo (adeno)tonsillectomy than girls is consistent with a recent study of children aged 4–15 years who were referred for (adeno)tonsillectomy [13]. That study found that older boys were more likely to choose conservative management rather than surgery when given the option. Furthermore, one of the greatest determinants for a preference for surgery, related to progress at school being affected. This observed age-sex distribution for (adeno)tonsillectomy, which reflects underlying age-sex specific ATI rates, also highlights a well described trend in the natural history seen for ATI and other childhood respiratory infections[24] [34].

Trends in (adeno)tonsillectomy cannot be ascribed to a single factor, as there are numerous potential explanations including the impact of guidelines and reviews, the views and attitudes of the referring family practitioners, the opinions of the otolaryngologists performing the operation and local referral pathways. Evolving pressures on parents to limit school absenteeism and restrictions about taking time off for working parents could also explain why operations are increasingly being performed before children commence school.

### Strengths and limitations of our study

This is among the first studies in England to depict the socio-demographic profile of children undergoing (adeno)tonsillectomy over time, using individual-level data from a large population-based sample. Our findings are unlikely to be a result of chance due to the large population size. However, there are a number of potential limitations. Firstly, large routine databases have potential inherent weaknesses, including missing or inaccurate data. However, the completeness of the socio-demographic variables within our data were >99% and the accuracy of HES has improved considerably over time [35]. Secondly, there may have been selection bias as HES only includes (adeno)tonsillectomies performed on the NHS and not the private sector. Approximately 16% of otolaryngology activity occurs in the private sector, so using HES data alone under-estimates the total number of operations performed [15]. Some parents in more affluent areas may seek private referral for (adeno)tonsillectomy and the effect of their inclusion might make this deprivation gradient less pronounced than it appears. Thirdly, the HES data does not include the actual indication for surgery. However, as described earlier, the age categories we selected are a proxy measure for the likely indication for surgery. Finally, we did not examine regional variations in deprivation which could indicate differences in resource allocation across the country.

In conclusion, we found that despite a reduction in (adeno)tonsillectomy rates among children aged 4–15 years, rates among pre-school children aged <4 years markedly increased over the last decade. Our findings suggest that the substantial increase in rates among children aged <4 years is likely to be a consequence of increased awareness and detection of OSAS, particularly in this younger age group, that has disproportionately affected those living in the most deprived areas. This highlights the need for national UK guidelines for the detection and management of OSAS in children, including criteria for performing (adeno)tonsillectomy. In view of escalating childhood obesity levels, the observed increase in (adeno)tonsillectomy rates among children aged <4 years and the deprivation gradient, a potential three-way association between OSAS, obesity and deprivation with (adeno)tonsillectomy still needs further investigation. Finally, the lack of long-term clinical and non-clinical outcomes data for children who undergo (adeno)tonsillectomy for recurrent throat infections and OSAS should be addressed as a priority, to enable clinicians to target those who are most likely to benefit from the operation.

## Supporting Information

Table S1 **Obstructive sleep apnoea syndrome and deprivation among children who underwent (adeno)tonsillectomy (aged 12–15 years).**
(DOCX)
