# Correction: Changing Indications and Socio-Demographic Determinants of (Adeno)Tonsillectomy among Children in England – Are They Linked? A Retrospective Analysis of Hospital Data

**DOI:** 10.1371/journal.pone.0112887

**Published:** 2014-10-31

**Authors:** The PLOS ONE Staff

There are errors in the Author Contributions. The correct contributions are: Conceived and designed the experiments: EK SS MS. Analyzed the data: EK. Wrote the paper: EK. Design and conception: EK SS MS. Data extraction: JM. Revising the manuscript critically for important intellectual content: EK AB JM MS SS. Guarantors of study: EK and SS.

In the Funding section, there is an error in the third sentence for the funding for author Sonia Saxena. The correct sentence is “SS is funded by a National Institute for Health Research Career Development Fellowship.”

Column 4 of Table 2 is incorrectly missing the age range. The correct age range is 8 to 11 years. The correct version of Table 2 can be viewed below.

**Table 2:** (Adeno)tonsillectomy by age group and sex in 2011/12 (N  =  27732).

	Age (years)				Total	P value*
SEX	<4	4 to 7	8 to 11	12 to 15		
Male (n)	3600	7322	1837	1164	13923	
%	63	55	40	28	50	
Female(n)	2120	6029	2722	2938	13809	P<0.001
%	37	45	60	72	50	
Total (n)	5720	13351	4559	4102	27732	
%	100	100	100	100	100	

Data source: Hospital Episodes Statistics data. *χ^2^ test for linear trend.

The 5^th^ sentence of paragraph 4 in the Results section incorrectly includes the phrase “in the ratio of.” The correct sentence is: “Conversely, among children in the 4–15 year age range, those who underwent surgery from the most deprived areas compared with the least deprived areas decreased between these study years and this was most pronounced among children aged 12–15 years (Tables 4 and 5).”
