# Supplementary material for: Changing Indications and Socio-Demographic Determinants of (Adeno)Tonsillectomy among Children in England – Are They Linked? A Retrospective Analysis of Hospital Data
Source: PLoS One. 2014 Aug 11;9(8):e103600. doi: 10.1371/journal.pone.0103600 (PMC4128587; doi:10.1371/journal.pone.0103600)
Supplement: Table S1 — Obstructive sleep apnoea syndrome and deprivation among children who underwent (adeno)tonsillectomy (aged 12–15 years). (DOCX) [file pone.0103600.s001.docx]

**Table S1: Obstructive sleep apnoea syndrome and deprivation among children who underwent (adeno)tonsillectomy (aged 12-15 years)**

| **Children aged 12-15 years who underwent (adeno)tonsillectomy** | | | | | | | | | |
| --- | --- | --- | --- | --- | --- | --- | --- | --- | --- |
|  | **OSAS diagnoses** | | | **Living in most deprived areas** | | | **OSAS AND living in most deprived areas** | | |
| **Year** | **n** | **%** | **P value*** | **n** | **%** | **P value*** | **n** | **%** | **P value*** |
|  | 42 | 0.7 | **P<0.001** | 1694 | 27.8 | P<0.01 | 13 | 0.2 | **P<0.001** |
| **2001/2** |  |  |  |  |  |  |  |  |  |
|  |  |  |  |  |  |  |  |  |  |
| **(n=6086)** |  |  |  |  |  |  |  |  |  |
|  |  |  |  |  |  |  |  |  |  |
|  | 183 | 4.5 |  | 1030 | 25.2 |  | 76 | 1.9 |  |
| **2011/12** |  |  |  |  |  |  |  |  |  |
|  |  |  |  |  |  |  |  |  |  |
| **(n=4095)** |  |  |  |  |  |  |  |  |  |
|  |  |  |  |  |  |  |  |  |  |

* χ^2^ test. OSAS – obstructive sleep apnoea syndrome

| Deprivation data >99% complete  Data source: Hospital Episodes Statistics data |  |  |  |
| --- | --- | --- | --- |
